# TSPO-induced degradation of the ethylene receptor RhETR3 promotes salt tolerance in rose (*Rosa hybrida*)

**DOI:** 10.1093/hr/uhae040

**Published:** 2024-02-15

**Authors:** Qingcui Zhao, Weikun Jing, Xijia Fu, Ruoyun Yang, Chunyan Zhu, Jiaxin Zhao, Patrick Choisy, Tao Xu, Nan Ma, Liangjun Zhao, Junping Gao, Xiaofeng Zhou, Yonghong Li

**Affiliations:** School of Food and Drug, Shenzhen Polytechnic, Shenzhen, 518055, Guangdong, China; Postdoctoral Innovation Practice Base, Shenzhen Polytechnic, Shenzhen, 518055, Guangdong, China; School of Food and Drug, Shenzhen Polytechnic, Shenzhen, 518055, Guangdong, China; Flower Research Institute of Yunnan Academy of Agricultural Sciences, Kunming, 650205, Yunnan, China; Department of Ornamental Horticulture, Beijing Key Laboratory of Development and Quality Control of Ornamental Crops, China Agricultural University, Beijing 100193, China; Department of Ornamental Horticulture, Beijing Key Laboratory of Development and Quality Control of Ornamental Crops, China Agricultural University, Beijing 100193, China; Department of Ornamental Horticulture, Beijing Key Laboratory of Development and Quality Control of Ornamental Crops, China Agricultural University, Beijing 100193, China; Department of Ornamental Horticulture, Beijing Key Laboratory of Development and Quality Control of Ornamental Crops, China Agricultural University, Beijing 100193, China; LVMH Recherche, F-45800 St Jean de Braye, France; LVMH Recherche, F-45800 St Jean de Braye, France; Department of Ornamental Horticulture, Beijing Key Laboratory of Development and Quality Control of Ornamental Crops, China Agricultural University, Beijing 100193, China; Department of Ornamental Horticulture, Beijing Key Laboratory of Development and Quality Control of Ornamental Crops, China Agricultural University, Beijing 100193, China; Department of Ornamental Horticulture, Beijing Key Laboratory of Development and Quality Control of Ornamental Crops, China Agricultural University, Beijing 100193, China; Department of Ornamental Horticulture, Beijing Key Laboratory of Development and Quality Control of Ornamental Crops, China Agricultural University, Beijing 100193, China; School of Food and Drug, Shenzhen Polytechnic, Shenzhen, 518055, Guangdong, China

## Abstract

The gaseous plant hormone ethylene regulates plant development, growth, and responses to stress. In particular, ethylene affects tolerance to salinity; however, the underlying mechanisms of ethylene signaling and salt tolerance are not fully understood. Here, we demonstrate that salt stress induces the degradation of the ethylene receptor ETHYLENE RESPONSE 3 (RhETR3) in rose (*Rosa hybrid*). Furthermore, the TspO/MBR (Tryptophan-rich sensory protein/mitochondrial benzodiazepine receptor) domain-containing membrane protein RhTSPO interacted with RhETR3 to promote its degradation in response to salt stress. Salt tolerance is enhanced in *RhETR3*-silenced rose plants but decreased in *RhTSPO*-silenced plants. The improved salt tolerance of *RhETR3*-silenced rose plants is partly due to the increased expression of *ACC SYNTHASE1* (*ACS1*) and *ACS2*, which results in an increase in ethylene production, leading to the activation of *ETHYLENE RESPONSE FACTOR98* (*RhERF98*) expression and, ultimately accelerating H_2_O_2_ scavenging under salinity conditions. Additionally, overexpression of *RhETR3* increased the salt sensitivity of rose plants. Co-overexpression with *RhTSPO* alleviated this sensitivity. Together, our findings suggest that RhETR3 degradation is a key intersection hub for the ethylene signalling-mediated regulation of salt stress.

## Introduction

Soil salinity is a widespread abiotic stress affecting plants and is becoming increasingly severe [1, 2]. Indeed, salt stress negatively affects the growth, development and yield of most crops [3]. Rose (*Rosa hybrida*) is one of the most important ornamental plants in the world. Rose cultivation relies entirely on irrigation; therefore, soil salinization is a major concern, dramatically decreasing the productivity and quality of rose plants and their flowers [4]. Plants have evolved and retained a series of regulatory mechanisms that allow them to withstand salt stress in their external environment, among which phytohormones, such as ethylene (ETH), abscisic acid (ABA), and salicylic acid (SA) play an important role [5–9]. Rapid accumulation of ABA under high salinity regulates the expression of salt-responsive genes, enabling plants to survive in unsuitable environments [10]. Exogenous application of SA leads to increased salt tolerance in maize (*Zea mays*) by accelerating photosynthesis performance [11].

Ethylene biosynthesis and signal transduction modulate salinity responses [12]. The ethylene signaling pathway comprises ethylene receptors and their downstream signalling components CONSTITUTIVE TRIPLE RESPONSE1 (CTR1), ETHYLENE INSENSITIVE2 (EIN2), ETHYLENE INSENSITIVITY FACTOR3 (EIN3), EIN3 BINDING F-BOXEs (EBFs), and others [13]. The ethylene receptor family in Arabidopsis (*Arabidopsis thaliana*) comprises five members, ETHYLENE RESPONSE1 (ETR1), ETR2, ETHYLENE RESPOSE SENSOR1 (ERS1), ERS2, and EIN4 [14–16], which act as negative regulators of the downstream signaling cascade. ETR1 and EIN4 inhibit seed germination under salt stress, while ETR2 stimulates germination, with ERS1 and ERS2 having no effect at this stage. The differences in seed germination under salt stress between *etr1* and *etr2* mutants are not caused by differences in ethylene production or ethylene sensitivity, but rather reflect changes in ABA signaling [17, 18]. Knockdown of MsETR2 abolished the improved salt tolerance mediated by ethylene in alfalfa (*Medicago sativa*) [19]. Salt stress induces the expression of the ethylene receptor gene *Histidine kinase 1* (*NTHK1*) in tobacco (*Nicotiana tabacum*) [20–23]; *NTHK1-*overexpressing plants showed lower ethylene sensitivity at the seedling stage, but increased salt stress tolerance [23]. In acorn squash (*Cucurbita pepo*), *ETR1A*, *ETR1B*, and *ETR2B* gain-of-function mutations enhanced ethylene insensitivity, but increased salt stress tolerance during seed germination and nutritive reproductive stages [24–26]. Therefore, ethylene receptors are clearly involved in the ethylene-mediated salinity stress response. In Arabidopsis, salt stress treatment modulates the 1-AMINOCYCLOPROPANE-1-CARBOXYLATE (ACC) SYNTHASE2 (ACS2) and ACS7 activity to induce ethylene production [27, 28]. The Arabidopsis mutants *ethylene overproducer1* (*eto1*), *eto2*, and *eto3* have increased ethylene production and higher tolerance under salt stress [29]. However, results from many previous studies have shown that ethylene-regulated salt stress responses in plants are complex, with both positive and negative regulation [12]. For example, overexpression of the wheat (*Triticum aestivum*) *ACC oxidase 1* (*ACO1*) gene in Arabidopsis significantly increased ethylene levels but elevated the salt sensitivity of transgenic plants as well [30]. Exogenous application of ethylene in rice (*Oryza sativa*) resulted in greater salt sensitivity [31]. Salt stress leads to a large and rapid production of reactive oxygen species (ROS) [32], resulting in oxidative stress that damages cellular structures and macromolecules such as DNA, lipids, and enzymes [33, 34]. In Arabidopsis, ETHYLENE RESPONSE FACTOR98 (ERF98; encoded by At3g23230) enhances salt tolerance through inducing ascorbic acid (AsA) biosynthesis and accelerating ROS scavenging [35].

**Figure 1:**
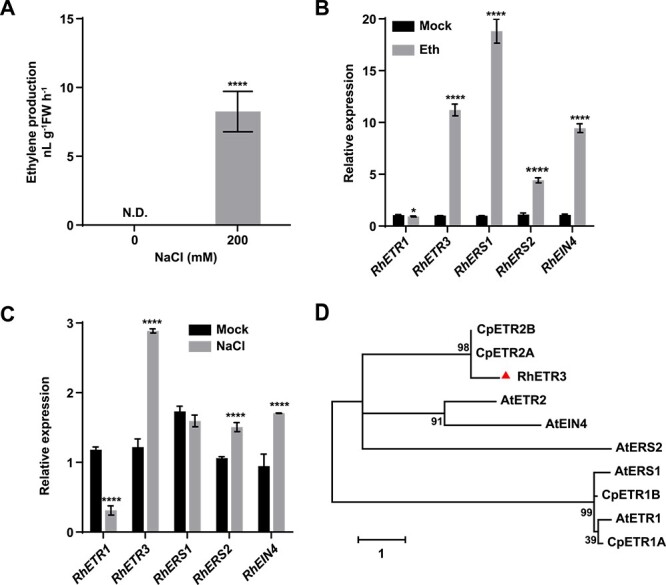
Expression of rose ethylene receptor genes in response to ethylene or salt treatment. (A) Ethylene production of rose leaves after salt stress. N.D., not detected. (B) Relative expression levels of ethylene receptor genes in response to 5 ppm ethylene for 3 hours. (C) Relative expression levels of ethylene receptor genes in response to 200 mM NaCl for 6 hours. (D) Phylogenetic analysis of ethylene receptors from rose and other plant species. The phylogenetic tree was reconstructed using MEGA 7.0.

The translocator protein of 18 kDa (TSPO) has a typical TspO/MBR domain that localizes to the membrane [36–38]. There is only one TSPO-related gene (*AtTSPO*) in Arabidopsis, encoding a protein that is involved in the response to multiple stresses [39, 40]. Salt stress causes AtTSPO to relocate from the endoplasmic reticulum (ER) to chloroplasts via its N-terminal extension. In addition, AtTSPO may function by regulating the transport of tetrapyrrole intermediates during salt stress treatment [40]. However, the underlying mechanism by which TSPO contributes to salt tolerance remains poorly understood.

In this study, we show that ethylene signaling regulates the salt stress response of rose through RhTSPO-mediated degradation of RhETR3, which triggers an increase in ethylene production and a decrease in H_2_O_2_ accumulation.

## Results

### The ethylene receptor RhETR3 negatively regulates salt tolerance in rose

Salt stress modulates the activity of ACS2 and ACS7 to induce ethylene production in Arabidopsis [27, 28]. Consistent with these published results, we observed increased ethylene production in rose leaves treated with salt stress imposed as 200 mM NaCl for 6 hours (Fig. 1A). To test the relationship between salt stress and ethylene in rose, we examined the expression of five rose genes encoding the upstream ethylene receptors under ethylene or salt stress treatment. Ethylene treatment inhibited the expression of *RhETR1* and promoted the expression of *RhETR3*, *RhRES1*, *RhERS2*, and *RhEIN4* (Fig. 1B). Salt treatment strongly and significantly increased the expression levels of *RhETR3* (Fig. 1C). Moreover, a phylogenetic analysis showed that RhETR3 is homologous to AtETR2, CpETR2A, and CpETR2B (Fig. 1D), which are all involved in salt stress responses, leading us to hypothesize that RhETR3 may be involved in the salt stress response of rose.

**Figure 2:**
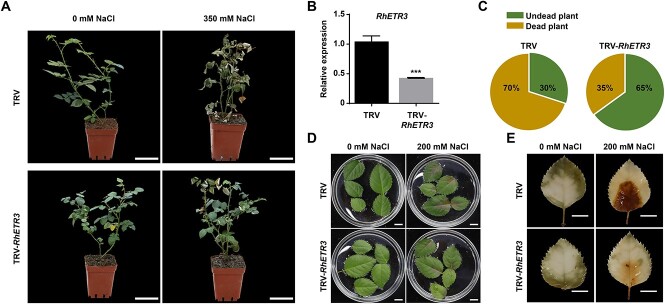
RhETR3 negatively regulates salt tolerance in rose. (A and C) Phenotypes of *RhETR3*-silenced plants in response to salt stress, imposed by watering plants with a 350 mM NaCl solution. Scale bars, 5 cm. (B) Relative *RhETR3* expression levels in TRV-infected control and TRV-*RhETR3* leaves. (D) Phenotypes of control TRV-infected and TRV-*RhETR3* leaves under salt stress. Scale bars, 1 cm. (E) 3,3′-diaminobenzidine (DAB) staining of control TRV-infected and TRV-*RhETR3* leaves under salt stress. Scale bars, 1 cm. Salt tolerance assays were performed three times (for each experiment, *n* = 15 for each line). Representative results are shown.

To determine the function of RhETR3 in salt stress, we silenced the *RhETR3* gene in rose using a virus-induced gene silencing (VIGS) approach (Fig. 2B). When rose plants silenced for RhETR3 via infiltration of a pTRV::*RhETR3* construct were irrigated alongside control plants infiltrated with the empty pTRV vector with 350 mM NaCl for 15 days, 65% of TRV control plants died. However, only 35% of *RhETR3*-silenced plants were dead under the same conditions (Fig. 2A, C). When rose leaves were treated with 200 mM NaCl treatment for 3 d, the damaged area of *RhETR3*-silenced leaf blades was significantly smaller than that of the TRV control (Fig. 2D). This finding was consistent with the staining pattern of mature leaves with 3,3′-diaminobenzidine (DAB) after salt stress (Fig. 2E), indicating that silencing of *RhETR3* improves plant salt tolerance and that RhETR3 negatively regulates the rose salt response.

### RhETR3 interacts with RhTSPO

We previously used a dual split-ubiquitin membrane-based yeast two-hybrid (MYTH) approach to screen for RhETR3-interacting proteins. We identified a TSPO/MBR family protein, encoded by the gene RU15274, as one of the candidate interacting proteins [41]. The predicted amino acid sequence of this protein contains a conserved TSPO/MBR domain, and a phylogenetic analysis showed that RU15274 is related to AtTSPO (Supplementary Fig. 1B); we therefore named this protein RhTSPO (Supplementary Fig. 1A). All TSPO-like proteins showed a high degree of sequence conservation (Supplementary Fig. 1A), suggesting that TSPO may have similar functions in different species. Previous studies have indicated that TSPO might be involved in abiotic stress responses. Moreover, expression of *TSPO* is induced by ABA, osmotic stress, and salt stress [23], prompting us to speculate that RhTSPO may act as a candidate interactor of RhETR3 and participate in the regulation of ethylene and salt stress signals together with RhETR3.

**Figure 3:**
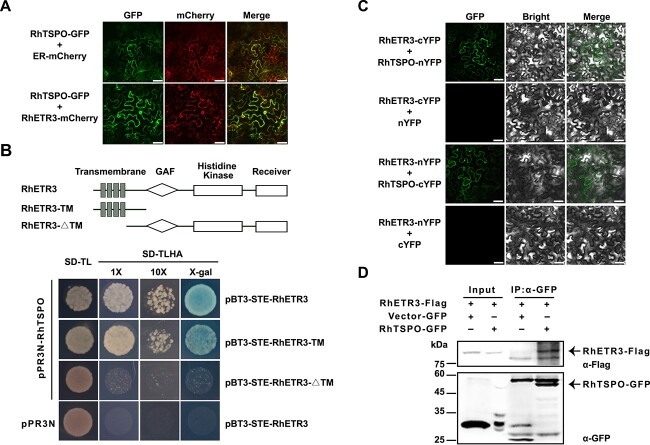
RhETR3 interacts with RhTSPO in vivo and in planta. (A) Co-localization of RhTSPO-GFP and RhETR3-mCherry in the leaves of *N. benthamiana* plants infiltrated with the indicated constructs. ER-mCherry was used as an endoplasmic reticulum (ER) localization marker. Scale bar, 50 μm. (B) Split-ubiquitin membrane-based yeast two-hybrid (MYTH) assay for the interaction of RhETR3 and RhTSPO. Top, diagram of RhETR3 and two truncated variants used in the assay. Bottom, MYTH assay for RhETR3 and RhTSPO. pPR3N with pBT3-STE-RhETR3 were used as negative control. (C) Bimolecular fluorescence complementation (BiFC) assay of the RhETR3–RhTSPO interaction in *N. benthamiana* leaves. The *RhETR3-cYFP* and *nYFP-RhTSPO* vectors were infiltrated into *N. benthamiana* leaves via Agrobacterium-mediated infiltration. R*hETR3*-nYFP and cYFP were used as negative control. Scale bars, 50 μm. (D) Co-immunoprecipitation (Co-IP) analysis of RhETR3 with RhTSPO *in vivo*. Total protein extracts (input) from *N. benthamiana* leaves infiltrated with the construcs *RhETR3-Flag* and *RhTSPO-GFP* were incubated with anti-GFP magnetic beads. Immunoblot analysis was performed using anti-GFP and anti-Flag antibodies. The detected proteins are indicated by arrowheads.

We determined that RhTSPO co-localizes with RhETR3 and an ER-localized marker in *Nicotiana benthamiana* leaves infiltrated with constructs encoding fusions to fluorescent proteins (Fig. 3A), which is consistent with a possible interaction between the two proteins. To delineate the domain involved in their protein–protein interaction, we conducted a targeted MYTH assay between RhTSPO and truncated RhETR3 and established that RhETR3 interacts with RhTSPO via the transmembrane domain (Fig. 3B). A bimolecular fluorescence complementation (BIFC) assay confirmed the interaction of RhTSPO and RhETR3 *in planta* (Fig. 3C). Moreover, we conducted a co-immunoprecipitation (Co-IP) assay by co-expressing *RhETR3-Flag* with *RhTSPO-GFP* in *N. benthamiana* leaves. Following total protein extraction from these leaves, we incubated the protein samples with ANTI-GFP Magnetic Beads. An immunoblot analysis of proteins co-precipitating with RhTSPO-GFP detected RhETR3-Flag, indicating that RhETR3 binds to RhTSPO (Fig. 3D). These results indicate that RhETR3 can physically interact with RhTSPO.

### RhTSPO positively regulates salt tolerance in rose

**Figure 4:**
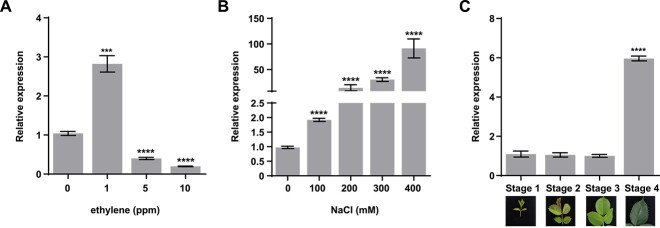
Expression patterns of *RhTSPO*. (A) Relative expression levels of *RhTSPO* in response to ethylene treatment. (B) Relative expression levels of *RhTSPO* in response to salt stress. (C) Relative expression levels of *RhTSPO* in rose leaves at different stages.

As RhTSPO interacted with RhETR3, we investigated the expression levels of *RhTSPO* under ethylene or NaCl treatments using reverse transcription quantitative PCR (RT-qPCR). Low ethylene levels (1 parts per million [ppm]) induced *RhTSPO* expression, whereas higher concentrations of ethylene (5 and 10 ppm) inhibited it (Fig. 4A). In addition, *RhTSPO* expression gradually increased with increasing NaCl concentration (Fig. 4B). Furthermore, *RhTSPO* expression was low in young leaves (stages 1–3) but high in mature leaves (stage 4) (Fig. 4C).

**Figure 5:**
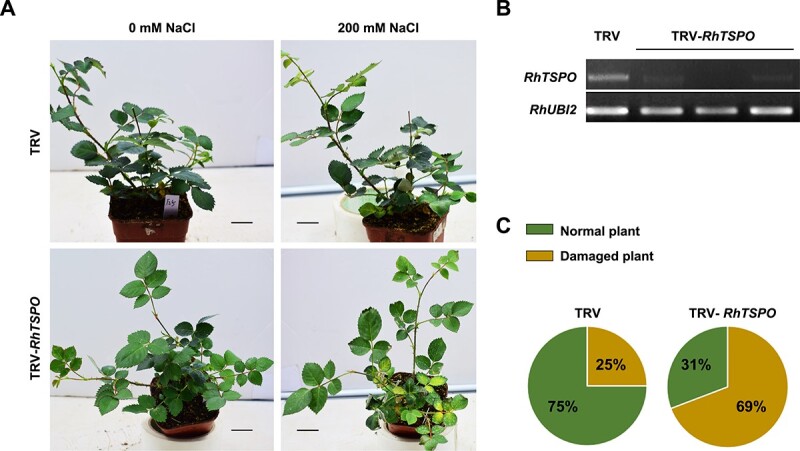
RhTSPO regulates plant tolerance to salt. (A and C) Phenotypes of *RhTSPO*-silenced plants in response to salt treatment. Scale bars, 2 cm. (B) RT-PCR validation of *RhTSPO*-silenced lines. The experiments were performed three times (for each experiment, *n* = 15 for each line). Representative results are shown.

Using a VIGS approach, we silenced *RhTSPO* in rose (Fig. 5B). Following salt treatment for 15 days, 69% of *RhTSPO*-silenced plants displayed typical injury symptoms, including leaf yellowing and wilting, as well as growth retardation. However, only 25% of TRV control plants showed a similar phenotype. These results indicate that silencing of *RhTSPO* compromised plant tolerance to salt (Fig. 5A, C).

**Figure 6:**
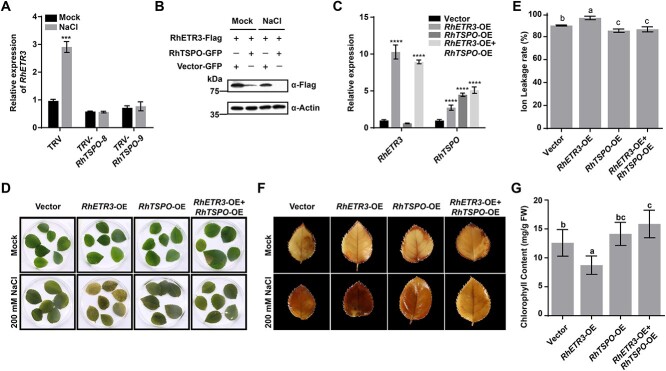
RhTSPO modifies the salt stress response by regulating RhETR3 protein levels in rose. (A) Relative expression levels of *RhETR3* in TRV and TRV-*RhTSPO* plants under control (Mock, 0 hour) conditions or after 6 hours of salt stress. (B) RhTSPO accelerates the degradation of RhETR3. *N. benthamiana* leaves expressing *RhETR3-Flag* and *RhTSPO-GFP* were infiltrated with no (0) or 200 mM NaCl for 6 hours before sample collection. RhETR3-Flag was detected by immunoblot analysis with an anti-Flag antibody. Actin served as a loading control. (C–G) Relative expression levels (C), phenotype (D), DAB staining (E), ion leakage (F), and chlorophyll content (G) of vector control*, RhETR3*-overexpression (OE), *RhTSPO*-OE, and *RhETR3* + *RhTSPO*-OE leaves under mock condition or salt stress. Salt tolerance assays were performed three times; representative results are shown. In G and E, different lowercase letters indicate significant differences (*P* < 0.05, Tukey’s multiple comparison analysis).

We also stably overexpressed *RhTSPO* in Arabidopsis (Supplementary Fig. 2A) and tested the response of these transgenic lines to salt treatment. As shown in Supplementary Fig. 2B, C, the germination rate of Arabidopsis Col-0 seeds significantly decreased as salt concentration in the medium increased, whereas transgenic lines overexpressing *RhTSPO* showed enhanced salt tolerance. In addition, only 7.4% of germinated Col-0 seedlings displayed healthy and green cotyledons under 100 mM NaCl treatment, while 20.4% and 56.1% of *RhTSPO*-OE#1 and *RhTSPO*-OE#4 seedlings, respectively, had green cotyledons (Supplementary Fig. 2D). These results suggest that RhTSPO has a positive regulatory effect on salt stress responses.

**Figure 7:**
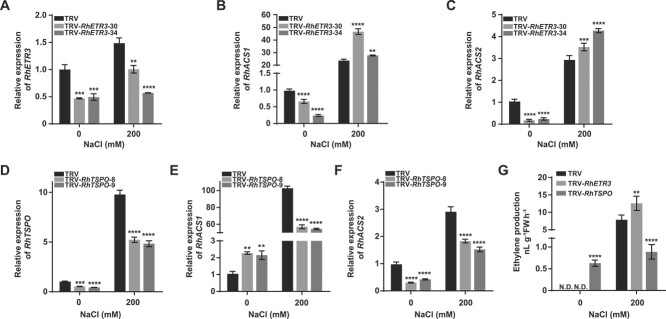
Effects of RhETR3 and RhTSPO on ethylene biosynthesis–related genes to regulate salt response in rose. (A) Relative *RhETR3* expression levels in TRV-infected control and TRV-*RhETR3* leaves with or without 200 mM NaCl treatment for 6 hours. (D) Relative expression levels of *RhTSPO* in TRV control and TRV-*RhTSPO* leaves with or without 200 mM NaCl treatment for 6 hours. (B, C, E, F) Relative expression levels of *RhACS1* and *RhACS2* in TRV, TRV-*RhETR3*, and TRV-*RhTSPO* plants with or without 200 mM NaCl treatment for 6 hours. (G) Ethylene production in the leaves of TRV and TRV-*RhETR3*, and TRV-*RhTSPO* plants with or without 200 mM NaCl treatment for 6 hours. N.D., not detected.

### RhTSPO mediates salt stress responses by promoting RhETR3 degradation

We speculated that RhTSPO may regulate the expression of *RhETR3* or affect RhETR3 abundance. We observed that the expression of *RhETR3* under control and salt-stress conditions is suppressed in *RhTSPO*-silenced plants (Fig. 6A), indicating that the expression of *RhETR3* is regulated by RhTSPO. However, the regulatory pattern between *RhETR3* and *RhTSPO* was not consistent with the salt stress response between them. To test the effect of RhETR3 on RhTSPO protein abundance, we co-expressed *RhETR3-Flag* and *RhTSPO-GFP* in *N. benthamiana* leaves and treated detached leaves with 200 mM NaCl for 6 hours. The salt stress treatment promoted the degradation of RhETR3-Flag, and the presence of RhTSPO-GFP exacerbated the instability of RhETR3-Flag (Fig. 6B). We conclude that salt stress induced the faster degradation of RhETR3 by RhTSPO.

We used the Super promoter to overexpress *RhETR3* (*RhETR3*-OE) or *RhTSPO* (*RhTSPO*-OE) and to co-overexpress *RhETR3* and *RhTSPO* (*RhETR3* + *RhTSPO*-OE) in rose leaves, using the empty vector as control. We then treated the plants carrying the infiltrated leaves with 200 mM NaCl for 4 days, after which the *RhETR3*-OE leaves turned yellow and white, whereas the empty vector control, *RhTSPO*-OE, and *RhETR3* + *RhTSPO*-OE leaves showed a more modest yellowing phenotype (Fig. 6C, D). Furthermore, an analysis of electrolyte leakage showed that the *RhETR3*-OE leaves exhibit a higher degree of damage than the *RhETR3* + *RhTSPO*-OE and control leaves under salinity stress (Fig. 6E). We also analysed ROS accumulation in control, *RhETR3*-OE, *RhTSPO*-OE, and *RhETR3* + *RhTSPO*-OE leaves using 3,3′-diaminobenzidine (DAB) staining under control and 200 mM NaCl conditions. The *RhETR3*-OE leaves produced a darker staining pattern than *RhETR3* + *RhTSPO*-OE and control leaves (Fig. 6F). In agreement with this observation, treatment with 200 mM NaCl, resulted in lower chlorophyll content in *RhETR3*-OE leaves compared to *RhETR3* + *RhTSPO*-OE and control leaves (Fig. 6G). These results indicate that the overexpression of *RhETR3* in rose plants leads to salt sensitivity, but co-overexpression or RhETR3 with *RhTSPO* counteracts this phenotype.

### The RhETR3–RhTSPO module regulates salt stress responses by altering ethylene production and the expression of the ethylene response factor *RhERF98*

In this study, we established that salt stress treatment induces ethylene production (Fig. 1A). Therefore, we examined the expression of the rose ethylene biosynthesis genes *RhACS1* and *RhACS2* [42, 43], which are involved in ethylene biosynthesis, in *RhETR3-* and *RhTSPO*-silenced plants. Under salt treatment, the expression of *RhACS1* and *RhACS2* was higher in *RhETR3*–silenced plants than that in TRV control plants (Fig. 7A–C), whereas we obtained the opposite result in *RhTSPO*-silenced plants (Fig. 7D–F). Consistent with the expression level results, ethylene content was higher in *RhETR3*-silenced leaves and lower in *RhTSPO*-silenced leaves compared to control plants under salt stress (Fig. 7G). These results suggest that the RhETR3–RhTSPO module is involved in the salt stress response by regulating ethylene production in rose.

**Figure 8:**
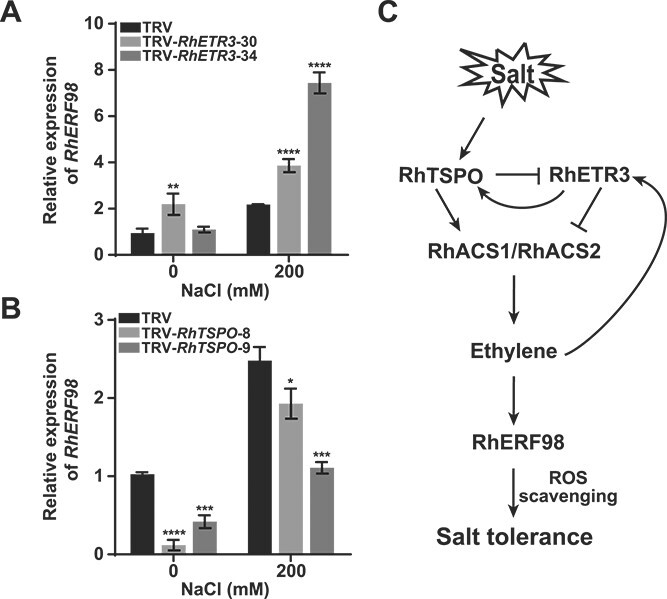
Effects of RhETR3 and RhTSPO on *RhERF98* expression to regulate salt response in rose. (A, B) Relative expression levels of the ethylene response gene *RhERF98* in TRV, TRV-*RhETR3*, and TRV-*RhTSPO* plants with or without treatment with 200 mM NaCl for 6 hours. (C) Proposed model of RhETR3–RhTSPO-mediated responses of rose plants to salt stress. When rose plants are exposed to salt stress, *RhTSPO* expression is rapidly upregulated. RhTSPO accelerates the degradation of RhETR3, upregulates the expression of the ethylene biosynthesis genes *RhACS1* and *RhACS2*, promotes ethylene production, and activates the expression of the ethylene response gene *RhERF98*, conferring salt tolerance by promoting ROS scavenging. Salt stress promotes ethylene production, and low concentrations of ethylene promote *RhTSPO* expression, further enhancing salt tolerance in rose plants.

As ethylene production is regulated by the RhETR3–RhTSPO module, we determined the expression of *RhERF98* in the leaves of *RhETR3-* and *RhTSPO*-silenced plants. We determined that *RhERF98* is an ethylene-responsive gene whose expression can be induced by salt stress. In Arabidopsis, AtERF98 binds to the promoter of the ASA biosynthetic gene *VITAMIN C DEFECTIVE1* (*VTC1*), increasing AsA biosynthesis, ROS scavenging, and salt tolerance [35]. After salt treatment, *RhERF98* expression was higher in *RhETR3*-silenced plants and lower in *RhTSPO*-silenced plants compared to control plants. This observation suggests that the RhETR3–RhTSPO module may alter the salt stress response of rose by controlling *RhERF98* expression (Fig. 8A, B). We also stably overexpressed *RhTSPO* or *RhETR3* in Arabidopsis and measured *AtERF98* expression under salt stress. *AtERF98* expression was lower in *RhETR3*-OE transgenic lines and higher in *RhTSPO*-OE transgenic lines than that in Col-0 (Supplementary Fig. 3A, B). In summary, salt stress activates a RhTSPO–RhETR3–RhACS1/2 signaling cascade that induces ethylene biosynthesis, increases *RhERF98* expression, and accelerates ROS scavenging to enhance salt tolerance in rose (Fig. 8C).

## Discussion

### Ethylene promotes the salt stress response in rose

Salt stress induces ethylene accumulation [12, 28]; however, ethylene-regulated salt stress responses in plants are complex, with both positive and negative regulatory networks [12].

The Arabidopsis ethylene overproduction mutants *eto1*, *eto2*, and *eto3* showed increased ethylene production and improved salt tolerance [29]. Moreover, salt stress modulates the activity of ACS2 and ACS7 to induce ethylene production in Arabidopsis [27, 28]. Ethylene promotes the expression of *MsERF11* and increase the germination rate of alfalfa seeds under salinity stress [19]. *NTHK1*-overexpressing tobacco plants are salt sensitive and have lower expression of ethylene biosynthesis genes and diminished ethylene production compared to wild-type plants [44].

Here, we provide evidence that silencing the ethylene receptor gene *RhETR3* in rose plants increases salt stress tolerance, expression of ethylene biosynthesis genes (*RhACS1* and *RhACS2*), and ethylene production (Fig. 7B, C, G). RhTSPO promoted RhETR3 degradation, and *RhTSPO*-silenced plants exhibited lower expression of *RhACS1* and *RhACS2*, lower ethylene production, and greater salt sensitivity, which are opposite phenotypes to those observed in *RhETR3*-silenced plants (Fig. 7E–G). These results suggest that salt stress induces ethylene production and that ethylene positively regulates the salt stress response in rose.

### The ethylene receptor RhETR3 negatively regulates the salt tolerance of rose in a TSPO-dependent manner

*AtETR1* is down regulated by salt stress, at both the transcript and protein levels [45]. Furthermore, analysis of loss-of-function mutants has shown that ETR1 inhibits seed germination and negatively regulates the salt stress response in Arabidopsis under salt stress [17]. In *C. pepo*, gain-of-function mutations in ethylene receptors (*CpETR1B*, *CpETR1A*, and *CpETR2B*) increased salt tolerance during seed germination and vegetative growth, suggesting that these ethylene receptors positively regulate the salt stress response. ETRs in Arabidopsis and *C. pepo* are involved in the salinity response by affecting the ABA signaling pathway, but not the ethylene signaling pathway [17, 18, 25]. Silencing of the rose ethylene receptor gene *RhETR3* not only increased the production of ethylene but also enhanced the salt tolerance of rose plants (Fig. 2A, D), whereas overexpression of *RhETR3* enhanced their sensitivity to salinity (Fig. 6D–G), indicating that RhETR3 negatively regulates the salt stress tolerance in rose and is dependent on the ethylene signaling pathway. We showed that salt stress induced the degradation of RhETR3, and its interacting protein RhTPSO accelerated this degradation (Fig. 6B). In addition, the rose salt sensitivity phenotype caused by overexpressing *RhETR3* can be diminished by overexpressing *RhTSPO* (Fig. 6D–G). However, the expression of *RhETR3* is upregulated by salt stress, and the salt stress-induced expression of *RhETR3* was suppressed in *RhTSPO*-silenced plants. We speculate that there is a feedback regulation of *RhETR3* expression in rose under salt stress, as increased *RhTSPO* expression during salt stress accelerated the degradation of RhETR3. These results indicate that RhETR3 affects the rose salt response in an RhTSPO-dependent manner.

### RhTSPO positively regulates plant salt tolerance in rose

TSPOs are conserved membrane proteins with a TspO/MBR domain. They have been extensively studied in mammals and bacteria, but their function remains largely unknown in plants [37, 38]. Previous studies have shown that TSPO may be involved in abiotic stress and that ABA, salt stress, and osmotic stress induce *TSPO* expression [39, 40]. Overexpressing *AtTSPO* increased salinity sensitivity during seedling growth in transgenic Arabidopsis [39]. In this work, we showed that salt stress induced the expression of *RhTSPO* in rose and that knockdown of *RhTSPO* conferred salt sensitivity. These results differ from previous reports, which presented that AtTSPO as a negative regulator of salt tolerance in Arabidopsis [40]. It is possible that TSPOs in different species may have evolved different regulatory mechanisms to cope with salt stress.

### The RhETR3–RhTSPO module affects the ethylene response factor gene *RhERF98* to regulate the salt response in rose

Fine-tuning of ethylene production and/or signaling can negatively or positively influence plant salinity sensitivity [12]. In tobacco, *NTHK1* overexpression enhanced salt sensitivity by affecting the expression of *NtACO3*, *NtERF1*, and *NtERF4* during salinity stress. In Arabidopsis, salt stress enhances AtERF98 expression and AtERF98 positively regulates plant salt stress responses. Here, we showed that RhETR3 and RhTSPO affect the production of ethylene and the expression of the ethylene response factor gene *RhERF98*. RhERF98 is a homolog of AtETR98, and AtERF98 positively regulates plant salt stress responses by increasing ascorbic acid (AsA) biosynthesis. We discovered that expression of the AsA biosynthesis genes *RhVTC1–1* and *RhVTC1–3* was higher in *RhETR3*-silenced rose plants but lower in *RhTSPO*-silenced rose plants after salt treatment (Supplementary Fig. 4), indicating that RhERF98 may affect AsA biosynthesis during salt stress treatment in rose. These results suggested that RhETR3 and RhTSPO are involved in salt stress responses, possibly by affecting ethylene signaling to regulate *RhERF98* expression, thereby altering AsA levels to affect ROS scavenging.

Here, RhTSPO positively regulated the transcription levels of *RhACS1* and *RhACS2*, while RhETR3 negatively regulated the expression of *RhACS1* and *RhACS2*. We speculate that the RhTSPO–RhETR3 module maintains a balance between the expression of *RhACS1* and *RhACS2* and ethylene biosynthesis in rose. When this balance is impaired, new regulatory mechanisms emerge to respond to changes. In our study, RhTSPO accelerated the degradation of RhETR3, while RhETR3 promoted the transcription of *RhTSPO*, which may be one of the regulatory mechanisms under such RhTSPO/RhETR3 imbalance. Under salt stress, the expression of *RhTSPO* is induced, and RhTSPO promotes the degradation of RhETR3. Therefore, the negative regulation on *RhACS1* and *RhACS2* expression by RhETR3 is weakened, while the positive regulation of *RhACS1* and *RhACS2* expression by RhTSPO is increased. Ultimately, the expression of *RhACS1* and *RhACS2* and ethylene biosynthesis increase, and the expression of *RhERF*98 leading to ROS clearance is induced, scavenging ROS and improving plant salt tolerance. In TRV-*RhETR3*, the pathway that inhibits *RhACS1* and *RhACS2* expression is blocked, and the expression level of *RhACS1* and *RhACS2* increases; *RhETR3*-silenced plants therefore exhibit greater salt tolerance. However, in TRV-*RhTSPO*, the pathway that promotes *RhACS1* and *RhACS2* expression is compromised, the expression of *RhACS1* and *RhACS2* increases, and the silenced plants show higher salt sensitivity. For the feedback regulation of *RhETR3* to promote *RhTSPO* expression, we speculate that excessive accumulation of *RhETR3* indicates an imbalance in TSPO/ETR3 regulation, and initiates a regulatory mechanism to degrade RhETR3. Under such conditions, inducing *RhTSPO* expression would help produce more RhTSPO proteins, which degrades RhETR3 (Fig. 8C). In conclusion, our findings revealed that the RhETR3–RhTSPO module is a critical node in the ethylene signaling–mediated salt stress response in rose.

## Materials and methods

### Plant materials and growth conditions

Rose plants (*R. hybrida* cultivar “Samantha”) were propagated by *in vitro* cultivation [46–48] and were transplanted into substrate 4 weeks after rooting. The temperature of the growth chambers was kept at 23°C ± 2°C, and the photoperiod was 16 hours light/8 hours dark with 70–80% relative humidity. Seeds of Arabidopsis (*A. thaliana*) accession Columbia (Col-0) were surface-sterilized with 0.6% (v/v) NaClO for 10 minutes and washed six times with sterile water. The seeds were then stratified for 3 days on Murashige and Skoog (MS) medium at 4°C in the dark before being transferred to growth chambers with the following conditions: 23°C ± 2°C, 70% to 80% relative humidity and a 16-hour light/8-hour dark photoperiod.

### Salt treatment

For rose plants, VIGS-mediated silenced lines and the TRV2 control plants were randomly divided into two groups. The plants were irrigated with 0, 200, or 350 mM NaCl and the phenotype was recorded after 2 weeks. Plants with withered stems and all their leaves having turned yellow were defined as dead. Plants with some yellow leaves and some growth inhibition were designated as damaged. For detached leaf assays, the leaves of rose plants were immersed in 200 mM NaCl, and the phenotype was observed after 3 days.

To determine *RhETR3* and *RhTSPO* expression in response to salt stress or ethylene treatment, rose leaves were treated with 0, 100, 200, 300, or 400 mM NaCl for 6 hours; plants were treated with 0, 1, 5, or 10 ppm ethylene for 3 hours.

For salt treatment, surface-sterilized Arabidopsis seeds were sown onto Murashige and Skoog (MS) medium alone or containing 50, 100, 150 or 200 mM NaCl. The germinated seeds and seedlings with green cotyledons were counted on the 7th and 10th day, respectively.

### RNA extraction and RT-qPCR

Total RNA was extracted from Arabidopsis seedlings with TRIzol® Reagent (Ambion) according to the manufacturer's instructions. Total RNA from rose leaves was extracted as previously described [49] and then reverse transcribed using HiScript II Q RT SuperMix (R223-01; Vazyme Biotech Co., Nanjing, China). qPCR was performed on a Real-Time PCR System (Applied Biosystems, CA, USA) with ChamQ SYBR qPCR Master Mix (Q331-01, Vazyme Biotech Co., Nanjing, China). *RhUBI*, *AtTUB*, and *AtUBC* were used as internal control transcripts. Primers used for RT-qPCR and RT-PCR are listed in Supplemental Table 1.

### Statistical analysis

Statistical analysis of the data was performed using GraphPad Prism 6.01 and IBM SPSS statistics. Paired data comparisons were analysed using two-sided Student's t-tests (*P < 0.05, **P < 0.01, ***P < 0.001, ****P < 0.0001), and Tukey's multiple comparison analysis was used to analyze differences between genotypes and treatments. All experiments were performed with at least three biological replicates and the error bars indicate the mean ± standard deviation (SD).

### Split-ubiquitin yeast two-hybrid assay

The pPR3N and the pBT3-STE vectors were used in this assay. The full-length coding sequence of *RhETR3* or sequences encoding truncated versions of RhETR3 without the transmembrane domain were cloned into pBT3-STE, while the full-length coding sequence of *RhTSPO* was cloned into pPR3N. The appropriate pairs of plasmids were co-transformed into yeast (*Saccharomyces cerevisiae*) NMY51 cells as described [50]. After 3 days of growth on synthetic defined (SD) medium -Leu-Trp, positive colonies were resuspended in 40 μl of sterile water, diluted 10×, and then plated onto SD-Leu-Trp-His-Ade medium alone or with β-galactosidase to test protein–protein interactions.

### Subcellular location analysis

The full-length *RhETR3* coding sequence was cloned into pSuper1300-mCherry, while that of *RhTSPO* was cloned in pSuper1300-GFP. Agrobacterium (*Agrobacterium tumefaciens*) strain GV3101 was transformed with each vector or empty vectors or with the P19 silencing suppressor. All cultures were resuspended in infiltration buffer (10 mM MES, [pH 5.6], 10 mM MgCl_2,_ and 200 mM acetosyringone) to a final OD_600_ of 1.5, or OD_600_ = 1.0 for the P19-harboring culture. The resuspended Agrobacterium cells were mixed at a ratio of 1:1:1 (v/v/v). After 3–5 hour, the bacteria were infiltrated into young *Nicotiana benthamiana* leaves. Fluorescence signals were observed by confocal microscopy (Olympus, FV3000, Japan) on the 3rd day after infiltration.

### Bimolecular fluorescence complementation assays

For bimolecular fluorescence complementation (BiFC) assays, the full-length *RhETR3* and *RhTSPO* coding sequences were cloned into the pSPYNE(R) vector to generate an N-terminal fusion to YFP (YNE) or the pSPYCE(M) vector to obtain an C-terminal fusion to YFP (YCE). The resulting *RhETR3-YNE* and *YCE* pair were introduced into Agrobacterium strain GV3101 for Agrobacterium-mediated infiltration as above; the construct RhETR3-YCE and the empty vector control YNE were used as negative control. The resuspended Agrobacterium cells were mixed at a ratio of 1:1:1 (v/v/v). After 3 to 5 hours, the bacteria were infiltrated into young *Nicotiana benthamiana* leaves. Fluorescence signals were observed by confocal microscopy at 1.5 days after infiltration.

### Co-IP assays and immunoblot analysis

*N. benthamiana* leaves co-expressing *RhETR3-Flag* and *RhTSPO-GFP* or *RhETR3-Flag* and *GFP* were collected for total protein extraction in native protein extraction buffer (50 mM Tris–HCl [pH 7.5], 10 mM Na_3_VO_4_, 5 mM EDTA, 5% [v/v] glycerol, 50 mM β-mercaptoethanol, 1 mM PMSF, 10 mM DTT, and 1× EDTA-Free Complete Protease Inhibitor Cocktail [Roche]) and 50 μM MG132 [51]. Thirty minutes on ice, centrifuge at 4°C for 10 minutes, aspirate the supernatant and repeat the previous step until no contaminants are present. Protein extracts were incubated with pre-washed BeyoMag™ ANTI-GFP Magnetic Beads (P2132) for 1 hour at room temperature. The beads were washed six times with Tris-buffered saline (TBS; 50 mM Tris–HCl, and 150 mM NaCl, pH 7.4) and boiled with 100 μL 2× SDS loading buffer (100 mM Tris–HCl [pH 6.8], 4% [w/v] SDS, 0.2% [w/v] bromophenol blue, 20% [v/v] glycerine, and 5% [v/v] β-mercaptoethanol), separated by SDS-PAGE, and immunoblotted with anti-GFP and anti-Flag antibodies after transfer to PVDF membrane.

For immunoblot analysis, protein extracts were separated on 10% (w/v) SDS-PAGE gels and then transferred to a PVDF membrane (Millipore) using transfer buffer (39 mM glycine, 48 mM Tris–HCl, and 20% [v/v] methanol) at 110 V for 60 minutes. Target proteins were detected using anti-GFP (Abmart), anti-Flag (Abclonal), or anti-actin (EASYBIO) antibodies, each at a 1:5000 dilution. Peroxidase conjugated anti-rabbit/mouse antibody (EASYBIO) was used at a 1:10 000 dilution as the secondary antibody.

### Plasmid construction and generation of transgenic plants

The full-length coding sequence of *RhTSPO* was cloned into the binary vector pSuper1300-GFP at the *Hind*III and *Spe*I restriction sites. The resulting vector was transformed into Arabidopsis plants using Agrobacterium-mediated transformation with Agrobacterium strain GV3101 [52].

For silencing of *RhETR3*, a 410-bp fragment containing a 135-bp fragment of the *RhETR3* 3′ untranslated region (UTR) was cloned into pTRV2 using the *Xba*I and *Sac*I restriction sites. For silencing of *RhTSPO*, a 400-bp fragment containing a 200-bp 3′ UTR fragment was cloned into pTRV2 using the *Xba*I and *Kpn*I restriction sites. Silencing of *RhETR3* and *RhTSPO* in rose plants by VIGS was performed as previously described [53].

*RhETR3* and *RhTSPO* were transiently overexpressed according to a previous method [54]. Briefly, the full-length coding sequence of *RhETR3* was cloned into pSuper1300-Flag using the *Sal*I and *Kpn*I restriction sites. For transient overexpression of *RhTSPO*, the pSuper1300-GFP-*RhTSPO* construct was used. Agrobacterium strain GV3101 was transformed with each vector or their corresponding empty vectors or with the P19 silencing suppressor. All cultures were resuspended in infiltration buffer as described above (subcellular location analysis), mixed, and then infiltrated into the leaves of rose plants of similar size and growth status. T The infiltrated plants were incubated in the light for 3 days at 23°C ± 2°C. *Vector*, *RhETR3*-OE, *RhTSPO*-OE, and *RhETR3*-OE + *RhTSPO*-OE leaves were incubated with 0 or 200 mM NaCl for 4 days, replacing the solution once a day.

### Ethylene measurement

Rose leaves were treated with 200 mM NaCl or treated with water only for 6 hours. The leaves were then collected and patted dry with absorbent paper, placed in a 25-mL gas collection bottle (two leaves per bottle), sealed, labelled, and left at room temperature. After 20 hours, the gas was mixed with a 2.5-mL syringe, 1 mL of the gas was immediately aspirated and injected into the a GC-2014 instrument (Shimadzu, Japan). The concentration of the compounds in the gas was detected by gas chromatography and recorded as C [55]. The leaves were removed from the bottle, and their fresh weight was measured and recorded as M. For each sample, five biological replicates were performed. Ethylene production was determined as follows (nL∙g^−1^ FW h^−1^) = [C × 25 / (M × 20)].

### DAB staining

To detect the content of H_2_O_2_, the 3,3′-diaminobenzidine (DAB) staining method was used [56]. Leaves incubated in water only or treated with 200 mM NaCl for 3 days were infiltrated with DAB staining solution, covered with filter cloth, vacuumed (0.8 Mpa for 5 minutes, three times) and left in the dark for 12 hours at room temperature. Stained leaves were transferred to destaining solution (ethanol/glycerol/acetic acid [3:1:1, v/v/v]) for 2 days, the destaining solution was changed every 12 hours, and photographs were taken to record the leaf DAB staining pattern.

### Determination of chlorophyll content

Leaf samples were blotted dry with filter paper and leaf veins were removed. For each sample, 0.1 g of leaf tissue was weighed; the leaves were cut into strips of 1 to 2 mm in width, placed in a 2-mL centrifuge tube, and incubated in the dark for 24 hours. The absorbance of all samples was determined at 649 and 665 nm on an A360 spectrophotometer. The total chlorophyll content (mg/g) = (18.16A_649_ + 6.63A_665_). Each treatment comprised five biological replicates.

### Determination of ion leakage rate

To determine the ion leakage rate of salt-treated leaves, two to three leaves were selected and placed in a 50-mL centrifuge bottle containing 20 mL deionized water and shaken at 28°C for 30 minutes at 200 rpm. The initial electrical conductivity (S1) of each sample was measured using a conductivity meter (DDBJ-350, LeiCi), the samples were boiled for 10 minutes, and the total electrical conductivity (S2) was measured after the samples had cooled to room temperature. Ion leakage = (S1 − S0) / (S2 − S0), where S0 is the deionized water conductance rate. Each treatment comprised five biological replicates.

### Sequence analysis

The amino acid sequences of the proteins related to ethylene receptor or RhTSPO were aligned using Clustal X2 software. The multiple sequence alignment was conducted using GeneDoc. The phylogenetic tree was generated with the Maximum Likelihood method in MEGA v7.1.

## Acknowledgements

This work was supported by the General Project of Shenzhen Science and Technology and Innovation Commission (grant 6020330006K0, grant 21K270360620), the National Natural Science Foundation of China (grant 32202530), Jinning Flower Industry Science and Technology Service Group (202204BI090022), Consult of Flower Industry of Jinning District (202204BI090022).

## Author contributions

Q.Z., W.J., Y.H., and X.Z participated in the design of this study. Q.Z., and W.J., J. F organized the results and charted. Q.Z., and X.Z wrote the manuscript. Y. L revised this manuscript. Other authors involved in related experiments. All authors read and approved the final draft of this article.

## Data availability

The data used to support the findings are available in the paper and in the supplementary materials that are available online.

## Conflict of interest statement

All authors declare no conflicts of interest.

## Supplementary Data

Supplementary data is available at Horticulture Research online.

## Supplementary Material

Web_Material_uhae040
