# Supplementary material for: TSPO-induced degradation of the ethylene receptor RhETR3 promotes salt tolerance in rose (Rosa hybrida)
Source: Hortic Res. 2024 Feb 15;11(4):uhae040. doi: 10.1093/hr/uhae040 (PMC11017515; doi:10.1093/hr/uhae040)
Supplement: Web_Material_uhae040 [file web_material_uhae040.zip › Supplementary Figure 20231124.docx]

**Supplementary** **Figure.** **S1 Gene features analysis of RhTSPO**.

(A) Alignment of TSPO protein sequences from rose and other plant species. CLUSTAL alignment of deduced amino acid sequences of RhTSPO and those of *Fragaria vesca subsp. vesca* (FvTSPO, XP_004290779.1), *Potentilla anserina* (PaTSPO, XP_050363373.1), *Prunus persica* (PpTSPO, XP_007200583.1), *Malus baccata* (MbTSPO, TQD95031.1), *Prunus yedoensis var. nudiflora* (PyTSPO, PQQ14544.1), *Pyrus × bretschneideri* (PbTSPO, XP_048442943.1), *Malus domestica* (MdTSPO, XP_008368988.1), *Prunus dulcis* (PdTSPO, XP_034227675.1), *Malus sylvestris* (MsTSPO, XP_050111632.1), *Arabidopsis thaliana* (AtTSPO, NP_566110.1). The TSPO/MBR domain is underlined. (B) Phylogenetic analysis of RhTSPO. The phylogenetic tree was reconstructed by MEGA 7.0. The scale bar represents the substitution rate per site.


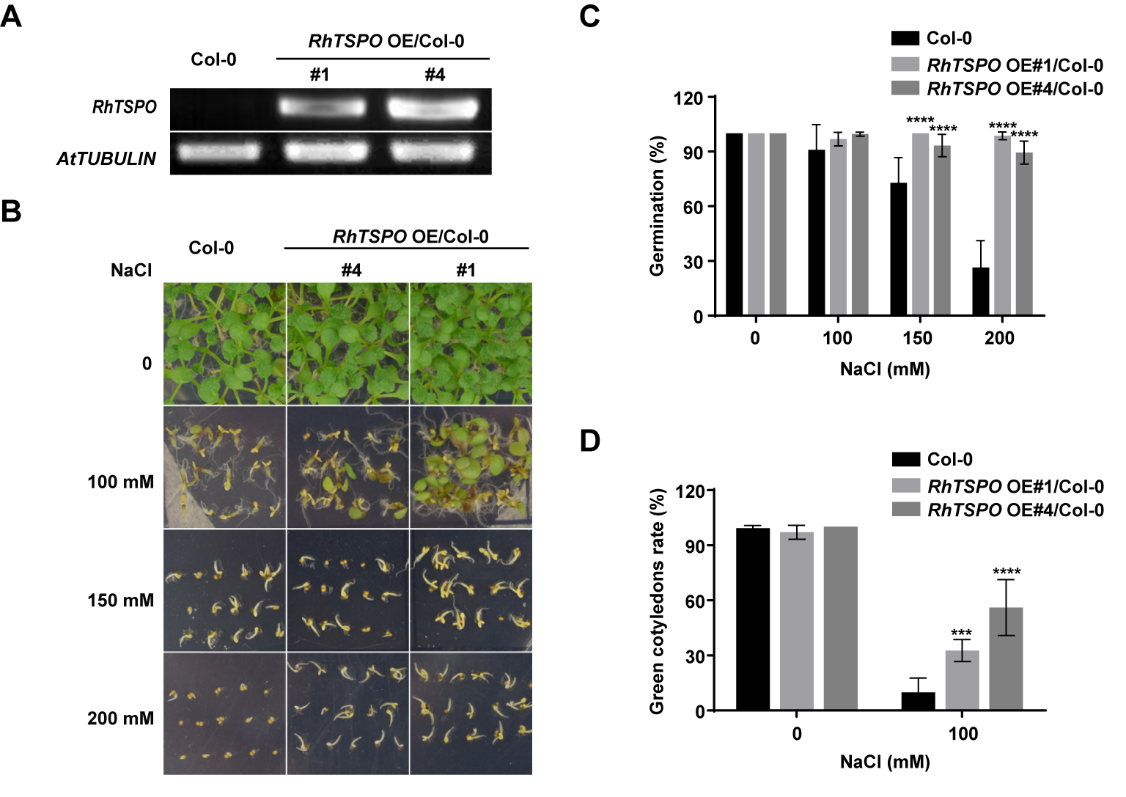


**Supplementary** **Figure. S2 RhTSPO enhances salt tolerance in Arabidopsis**.

(A) RT-PCR validation of *RhTSPO*-overexpression lines. (B and C) Overexpression of *RhTSPO* enhances seed germination on MS medium containing NaCl (100, 150, and 200 mM). (D) Percentage of seedlings with green cotyledons growing on MS medium containing NaCl. The experiments were performed six times (for each experiment, *n* = 30 for each line). Values are means ± SE. Asterisks indicate significant differences according to Student’s t-test (***P < 0.001, ****P < 0.0001).


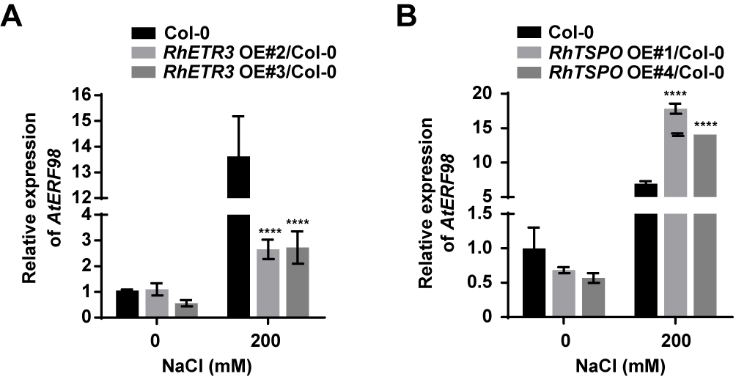


**Supplementary** **Figure. S3 Effects of RhETR3 and RhTSPO on *AtERF98* expression to regulate salt response in Arabidopsis.**

Expression of the ethylene response gene *AtERF98* in Col-0, *RhETR3*-overexpression (OE) (A) and *RhTSPO*-OE (B) seedlings after 6 h of salt stress. Values are means ± SD (*n* = 3). Asterisks indicate significant differences according to Student’s t-test (**P < 0.01, ***P < 0.001, ****P < 0.0001).


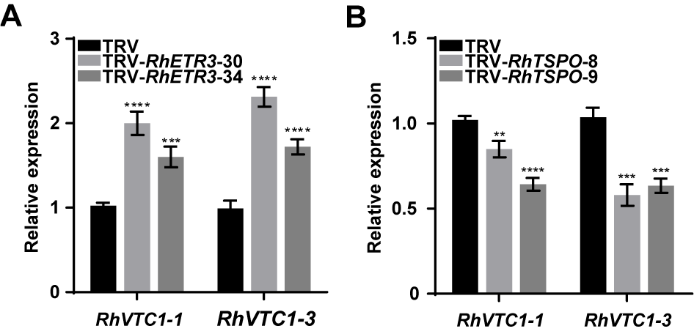


**Supplementary** **Figure. S4 Effects of RhETR3 and RhTSPO on the expression of ascorbic acid (AsA) biosynthesis genes to regulate the salt response in Arabidopsis.**

Expression of genes related to AsA biosynthesis in the leaves of TRV, TRV-*RhETR3*(A), and TRV-*RhTSPO* (B) plants after 200 mM NaCl for 6 h. Values are means ± SD (*n* = 3). Asterisks indicate significant differences according to Student’s t-test (**P < 0.01, ***P < 0.001, ****P < 0.0001).

**Supplementary Table S1** Primers used in this study

| **Primer names** | **Primer sequence (5'-3')** |
| --- | --- |
| *RhETR3*-BIFC-F | CGGGATCCATGTTAAAGGCATTAGCATCTGGGC |
| *RhETR3*-BIFC-R | CGGGGTACCCACAATTTTGTTTGCCTGCACCA |
| *RhTSPO*-BiFC-F | GCTCTAGAATGGATTCCCAAAACCTCAAGCA |
| *RhTSPO*-BiFC-R | CGGGGTACCGAGATACACAAGCTTAAGATTTACAACG |
| *RhTSPO*-PR3N-F | TAACAAGGCCATTACGGCCATGGATTCCCAAAACCTCAAGCA |
| *RhTSPO*-PR3N-R | AACTGATTGGCCGAGGCGGCGAGATACACAAGCTTAAGATTTACAACG |
| *RhETR3*-STE-F | ATTAACAAGGCCATTACGGCCTTAAAGGCATTAGCATCTGG |
| *RhETR3*-STE-R | AACTGATTGGCCGAGGCGGCCCCCACAATTTTGTTTGCCTG |
| *RhETR3*-STE-TM R | AACTGATTGGCCGAGGCGGCCCCCACAATTTTGTTTGCCTGC |
| *RhETR3*-STE-△TM F | ATTAACAAGGCCATTACGGCCTCGAATGCTTACCCAAGAGATTCG |
| *RhETR3*-1300-F | ACGCGTCGACATGTTAAAGGCATTAGCATCTG- |
| *RhETR3*-1300-R | GGGGTACCTTACTTGTACAGCTCGTCCATGC |
| *RhTSPO*-1300C-F | ACGCGTCGACATGGATTCCCAAAACCTCAAGC |
| *RhTSPO*-1300C-R | GACTAGTAGAGATACACAAGCTTAAGATTTACAAC |
| *RhETR3*-TRV2-F | GCTCTAGA TTTCTGCCATTGGTCCCT |
| *RhETR3*-TRV2-R | GCGAGCTC TCTAACTGTGAATGTTGGTGTAA |
| *RhTSPO*-TRV2-F | GCTCTAGAGCCCTTCTGGTTCCCGC |
| *RhTSPO*-TRV2-R | GGGGTACCGAAGATCTTAATGGCCATAAGAGAGTG |
| *RhUBI* QF | GCCCTGGTGCGTTCCCAACTG |
| *RhUBI* QR | CCTGCGTGTCTGTCCGCATTG |
| *RhETR1* QF | ACGGGGTCAGCAGGTCAGCGA |
| *RhETR1* QR | TCCATCCATA CCAACCCTCA TGCAG |
| *RhETR3* QF | TGGTATGTGTTTCGGCGTCT |
| *RhETR3* QR | AAGTAGGCCACCGCAATCAA |
| *RhERS1* QF | TGCTCAGCCCAGAAATGGATCCAATCGAGTA |
| *RhERS1* QR | ACGCGGATTT GTTGAAGCTA AACCGT |
| *RhERS2* QF | TAAACTTT TTGTCTCTGT GTGG |
| *RhERS2* QR | CTAACTTACAAATTCTTGATTGC |
| *RhEIN4* QF | CAAC AAATGTGGAG AACAGGT |
| *RhEIN4* QR | GATTGATTGGATTCATGGACAT |
| *RhTSPO* QF | TCTGTGGGGCTTTCGTCTG |
| *RhTSPO* QR | GCAACCCAAGCCAAACAAGGC |
| *RhACS1* QF | ACGGTCATGTTTTGCCTGGC |
| *RhACS1* QR | GTGAGTGTGTCTCTGTCTACGGT |
| *RhACS2*QF | TGAATCTCGTGTGCCTTGCT |
| *RhACS2* QR | GGCCGTAAAGCTTCAACTGGA |
| *RhERF98* QF | CAGGCATGGAAGAAGTGCGT |
| *RhERF98* QR | ACGGATGCTTGGAATCTCGGAG |
| *RhVTC1-1* QF | CGAAACTGCCCTTCACCAAAGCA |
| *RhVTC1-1*QR | GCCAGAACCACTTCGGTCACA |
| *RhVTC1-3* QF | ATGAGCTTAGAGTGCCGGTG |
| *RhVTC1-3*QR | GCCTTCCCGGTGACTGGATAC |
| *AtERF98* QF | CAAGGGCTTATGACCGAGCAG |
| *AtERF98* QR | CATAAGGAGGGCGAAGCGAG |
| *RhERF98* QF | CAGGCATGGAAGAAGTGCGT |
| *RhERF98* QR | ACGGATGCTTGGAATCTCGGAG |
| *RhVTC1-1* QF | CGAAACTGCCCTTCACCAAAGCA |
| *RhVTC1-1*QR | GCCAGAACCACTTCGGTCACA |
